# Psychotherapy in Europe

**DOI:** 10.1177/0952695118808411

**Published:** 2018-12-12

**Authors:** Sarah Marks

**Affiliations:** Birkbeck, University of London, UK

**Keywords:** clinical epistemologies, European history, geographies of science, psychoanalysis, psychotherapy

## Abstract

Psychotherapy was an invention of European modernity, but as the 20th century unfolded, and we trace how it crossed national and continental borders, its goals and the particular techniques by which it operated become harder to pin down. This introduction briefly draws together the historical literature on psychotherapy in Europe, asking comparative questions about the role of location and culture, and networks of transmission and transformation. It introduces the six articles in this special issue on Greece, Hungary, Yugoslavia, Russia, Britain and Sweden as well as its parallel special issue of *History of Psychology* on ‘Psychotherapy in the Americas’. It traces what these articles tell us about how therapeutic developments were entangled with the dramatic, and often traumatic, political events across the continent: in the wake of the Second World War, the emergence of Communist and authoritarian regimes, the establishment of welfare states and the advance of neoliberalism.

Psychotherapy was quintessentially an invention of European modernity. But as the 20th century unfolded, and we trace how psychotherapy crossed national and continental borders, its goals and the particular techniques by which it operated become harder to pin down. Perhaps the clearest thing we can learn from looking comparatively across the European continent – and beyond – is that practices and theories proliferated, migrated and adapted to local needs and contexts, as the six articles in this special issue show. The object upon which different forms of therapy claimed to be acting – whether it was the mind, the body, the unconscious, behaviour, morality or rationality, or something even more transcendental – was also contingent upon particular intentions and traditions. These were frequently at a scale much smaller than that of the nation – although the state, its health systems, social norms and collective experiences did shape praxis and provision in many countries, especially in the post-war period. This issue, and its parallel special issue of *History of Psychology* on ‘Psychotherapy in the Americas’, edited by Rachael Rosner, brings together new research to expand the geographical reach of our field. It asks questions about the role of location and culture, and the nature of transmissions and transformations, as concepts and personnel moved across and between continents, and it furthers the agenda of this journal to showcase recent historical research on psychotherapy, including last year’s special issue on ‘Psychotherapy in Historical Perspective’ (Marks, 2017).

It also draws on a rich literature in science studies on the ‘spatial turn’ (Secord, 2004), and builds on an emerging historiography in the psy-disciplines that pay attention to regions often considered to be on the ‘periphery’, drawing on archival and printed sources in languages beyond the more familiar Germanophone, Francophone and Anglophone record (Baker, 2012; Gjuričová, 2018; Madsen, 2018; Mühlberger, 2012; Raikhel and Bemme, 2016; Savelli and Marks, 2015). The articles in both issues also uncover the degree to which histories of psychotherapy were entangled with transformative and traumatic political events: in the wake of the Second World War, the emergence of Communist and authoritarian regimes, the establishment of welfare states and the advance of neoliberalism as the 20th century gave way to the 21st. Specifically, they open up new understandings of the place of psychotherapies in Greece, Hungary, Yugoslavia, the Russia, Britain and Sweden. But what has already been said about the rest of Europe?

The literature on France posits an alternative origin story to the familiar narrative that Vienna was the birthplace of psychotherapy by virtue of Freud’s invention of the psychoanalytic ‘talking cure’. Gladys Swain and Marcel Gauchet trace the emergence of a range of psychotherapeutic practices from the early nineteenth century ‘moral treatment’ of Pinel and the Tuke family (Gauchet and Swain, 1994). Mark Micale and Jacqueline Carroy also show how hypnosis, suggestion and trauma discourse opened up the cultural terrain for psychotherapies – including psychoanalysis – to flourish in the 20th century (Carroy, 1991; Micale, 2001). Trauma has been a recurrent motif in the literature more broadly, with others arguing that devastation of the Great War spurred military psychiatrists such as W.H.R. Rivers to look to psychoanalysis for answers in treating soldiers with war neurosis, giving it a more credible place in British practice in the interwar period (Leese, 2001). More recent scholarship on psychotherapy in France has also uncovered the late reception of behaviour therapies, in part because of the popularity of psychoanalysis among clinicians and intellectuals at large, but also because of the sceptical associations it provoked in relation to American culture, and technologies of mind control (Amouroux, 2017).

This raises the question of how certain therapies came to be viewed as coercive, and whether there were cases of historical collusion between practitioners and the darker aspects of the state. Geoffrey Cocks showed how non-Jewish psychotherapists managed to negotiate protection within the walls of the Göring Institute, with substantial economic backing from the Nazi party and Reich government (Cocks, 1985). Of course, such cases cannot be confined to Nazi Germany: recent literature has also unearthed uncomfortable links between repressive governments, security services and psychotherapeutic professions in the Cold War, and under authoritarian regimes in Latin America (Damousi and Plotkin, 2012; Ffytche and Pick, 2016; Rubin, Mandelbaum and Frosh, 2016). This reminds us that psychotherapy, as with psy- and medical professions, has also been a site of abuse – both at the level of institutions and the state, as well as cases of more quotidian, interpersonal coercion. Articles in this issue by Åsa Jansson, Julia Gyimesi and Aleksandra Brokman foreground cases where these ethical tensions are apparent. How these histories have (or have not) impacted on ethical debates within psychotherapy, and its regulation, still remains an open question.

After the Second World War, psychosomatic approaches came to have a dominant position on both sides of the Berlin Wall, albeit in different ways, but this ensured that psychotherapy had a place in both East and West German medicine. In the German Democratic Republic, faced with a large number of patients out of work due to apparently psychogenic physical ailments, doctors turned to the autogenic self-hypnosis techniques of the Jena physician, J.H. Schultz, reinscribing them in the language of Pavlovian conditioning (Geyer, 2011; Marks, 2018). In the Federal Republic of Germany, psychoanalysts managed to maintain a foothold by adapting their practice to a specific psychosomatic subspecialty of medicine that had become part of the establishment from the late 1940s, funded by the insurance system. The medical community’s tolerance of this – in spite of a long-standing scepticism about psychoanalysis – might be read in part as compensatory for the ‘excesses’ of biological psychiatry under Nazism (Roelcke, 2004).

Post-war psychotherapy in Europe and elsewhere was also haunted by the shadow of the Holocaust. This included the generation of new clinical approaches by survivors such as Victor Frankl, who drew on his experiences to establish the existentially-informed ‘Logotherapy’ after his return to Vienna (Carter, 1992). Substantial attention has been paid to the phenomenon of the intergenerational transmission of trauma and its treatment, with psychotherapeutic collaborations emerging in Poland and the Czech Republic as well as in Britain, Germany and elsewhere (Bomba, 2013; Frosh, 2013).^1^

These parallel special issues, and last year’s predecessor in this journal (Marks, 2017), make a point of expanding the historiography of psychotherapy beyond psychoanalysis, to include practices emerging from religious and spiritual traditions, cognitive and behavioural approaches, hypnotherapies and psychosomatic suggestion, mind cure, art therapies and psychotherapeutics carried out under the influence of psychedelics, among many other approaches (Cummings, 2017; Dyck and Farrell, 2018; Kirkham, 2017; Rosner, 2018a, 2018b). But this is not to underplay the significance of psychoanalysis on a global scale and in Europe, across both West and East, not to mention its substantial and enduring dominance in parts of Latin America (Dagfal, 2018; Ffytche and Pick, 2016; Marks, 2015; Savelli, 2013). For the Spanish case, scholarship on psychotherapy focuses on the emergence of psychoanalytic concepts via Freud’s reception among neuropsychiatrists in the 1920s and how these became popularised by Emilio Mira through a Barcelona-based journal (Allodi, 2012; Carles *et al.*, 2000; Mir, 2011; Mühlberger *et al.*, 2015). Similarly for Italy, the emerging literature focuses on the way the profession of clinical psychology received psychoanalysis, and how this informed their practice (Cimino and Foschi, 2018).

Returning to Britain, psychoanalytic thought in the 1940s and its place in the culture of post-war settlement has been a site of keen historical interest since the 1990s – especially in relation to the Tavistock Clinic. This includes close attention to attachment theorists, such as John Bowlby and Donald Winnicott, and how their ideas were shaped by notions of democratic selfhood, in turn being taken up in the construction of the welfare state itself (Alexander, 2016; Rose, 1991; Shapira, 2013). This concern with rebuilding Europe through nurturing a liberal psyche was also a response to the horrors of Nazism – with Tavistock clinicians such as Wilfred Bion and S.H. Foulkes experimenting with group approaches, which would go on to inform the development of group psychotherapies worldwide (Pick, 2012). This post-war, psychoanalytically inspired approach appears to have been a significant export from Britain. Danae Karydaki and Mat Savelli demonstrate how therapists in Greece and Yugoslavia respectively travelled for training and seminars to the Tavistock Clinic. They, in turn, informed the development of practices in both countries as they established their own place in newly organised healthcare systems. Similar trajectories have been noted elsewhere, including the adoption of group psychotherapies in Spain (Mir, 2011). Mat Savelli’s article bolsters the literature that shows the legacies of psychoanalysis in the Communist East as an underground practice, which also quietly informed new developments in psychotherapy that were used in more mainstream, state-sponsored settings (Antić, 2017; Buda *et al*., 2009; Leuenberger, 2001; Marks, 2015; Savelli, 2013). Savelli argues that the focus on group approaches was not due to ideological pressure in favour of privileging social collectivity, but was largely a result of clinicians’ familiarity with the practices of Bion and Foulkes from their training abroad.

In Greece, even though psychoanalysis had achieved an intellectual following from much earlier in the century, it wasn’t until after the fall of the military junta in 1974, and the foundation of a social democratic nationalised healthcare system, that psychoanalytic psychotherapies began to be offered in an organised capacity, albeit with limitations. Many of these therapists had spent time prior to this abroad, also notably in London. Karydaki links the place of psychoanalysis in Greek society to long-term experiences of national trauma, both as a consequence of Nazi occupation and civil war. In her recent book, *Therapeutic Fascism*, Ana Antić has drawn a connection between the psychiatric profession and their role in treating the trauma of war in Yugoslavia – and the use of psychotherapeutically inspired interventions by both right and left as a means of re-education (Antić, 2017). The psy-professions and psychiatric knowledge have clearly often played a clinical role in the aftermath of trauma in Europe, for better or worse, as well having entered societal discourses about coming to terms with the experience of violence (LaCapra, 1998).

A key theme brought forward by many of the authors in the issue is that of transnational exchange, and the need for historians of psychotherapy to acknowledge the international nature of intellectual, scientific and medical culture in the modern world. Transatlantic connections are also traced in the articles in our parallel special issue on psychotherapy in the Americas, which show the take-up and adaptation of therapeutic ideas from Europe into Latin America and Canada – particularly the appropriation of French concepts into Argentinian therapeutic culture, as well as British and Canadian connections in psychedelic experimentation (Dagfal, 2018; Dyck and Farrell, 2018). Other authors have traced the complex processes by which Central European psychoanalysts resettled in the United States, often through forced migration after the rise of fascism on the continent, and became increasingly influential in American psychiatry after the Second World War (Erös, 2016; Hale, 2000).

In this issue, knowledge transfer in the opposite direction comes to the fore. Åsa Jansson shows how dialectical behaviour therapy (DBT), initially developed by Marsha Linehan at the University of Washington to treat patients with Borderline Personality Disorder, was imported into the Swedish psychiatric context. Matthew Drage also looks at an approach that, along with DBT, is considered to be one of the ‘third wave’ cognitive behavioural therapies: mindfulness-based CBT (MBCBT). Like DBT, this modality was developed and promoted by a particular individual – in this case, Jon Kabat-Zinn in the United States. Drage’s analytic framework draws on Weberian ideas of charisma to trace how the ‘transmission’ of MBCBT occurred through networks of key actors in Britain from the 1970s.

As Mat Savelli argues in his article, it is crucial that attention is paid to the wider, comparative context and the degree to which actors were able to access traditions from elsewhere via conferences, correspondence, the procurement of texts and training abroad. Without such analyses, it can be all too easy to attribute developments to monocausal local specificities, such as ideological factors, or to assume continuities from an earlier period. If we unpick the networks at play in shaping therapeutic practice, and look at simultaneous trends in other parts of Europe and elsewhere, we can better ascertain the interaction between the local and the global. This is especially key for Eastern Europe where there were idiosyncratic developments in particular localities, but where intellectual and clinical communities were not hermetically sealed from the wider world (Aleksandrowicz, 2009; Buda *et al*., 2009; Marks, 2015; Matza, 2018; Raikhel and Bemme, 2016). Continuities, and actual personal connections, across East and West were sometimes more pronounced than might appear at first glance.

The impact of transnational networks is unquestionable and no clinical culture operated in an intellectual vacuum, divorced from external influence in the twentieth century. But psychotherapeutic methods were not always imported wholesale from abroad – sometimes they had to be adapted, as in Yugoslavia and Greece, to the constraints of local health systems. As Aleksandra Brokman shows for the Soviet Union, ‘minor psychotherapy’ – a suggestive technique to facilitate the success of medical procedures and improve psychosomatic illnesses – developed in a way quite different to Western techniques, although some aspects were shared with hypnosis and suggestion common across Europe from the late nineteenth century. Importantly, it became grafted on to long-standing power dynamics in terms of paternalism within the doctor–patient relationship in Russian clinical culture. According to those promoting it, psychotherapeutic suggestion actively capitalised on the authority of the doctor figure as a way of manipulating the placebo effect in the interests of the patients’ health.

Julia Gyimesi, writing on the case of the hypnotherapist Ferenc Völgyesi in Hungary, also illustrates the persistence of suggestion as a mode of treatment in the East European region. This case is demonstrative of the ease with which, during the Communist period, it could be defended by recourse to Pavlovian theories of higher nervous activity, with suggestive words apparently acting as a form of conditioned reflex. Researchers have documented how hypnosis, autogenic relaxation techniques, and other psychotherapies based on suggestion, remained popular in both the clinical and popular realms across the Soviet sphere, usually building on traditions that preceded the establishment of Communism in the region, but perhaps benefitting from an ideological climate that privileged approaches which resonated with ‘Pavlov’s teachings’ (Brokman, 2018; Geyer, 2011; Lauterbach, 1984; Marks, 2018). Julia Gyimesi’s and Matthew Drage’s articles, along with those in the parallel special issue on the relationship between therapeutics and spiritism in Brazil and Cuba, and mind cure and mysticism in the United States, remind us that the history of psychotherapy has long been entangled with religious and esoteric beliefs (Fachinetti and Jabert, 2018; Lambe, 2018; Schmidt, 2018).

Finally, Drage’s article on MBCBT and Jansson’s piece on DBT both argue that these technologies of the self emerged concurrently with the neoliberal age, drawing on the well-known work of Nikolas Rose on the interrelationship between the psy-disciplines and governmentality (Rose, 1991). Jansson charts how the rise in self-regulatory outpatient therapies for Borderline Personality Disorder occurred in tandem with the decline of the traditional Scandinavian model of the welfare state – and particularly the closure of inpatient psychiatric facilities. Ole Jacob Madsen has noted, simultaneously, the process of the ‘psychologization’ of cultural discourse in Norway, arguing that there are ethical implications thrown up when therapeutic techniques and modes of self-understanding migrate outside of the clinical setting (Madsen, 2018). Matthew Drage reminds us that the rise of mindfulness should be considered within the ‘close-knit matrix of professional expertise, measurement, metricisation, institutional discipline and surveillance which, tied to an ethic of personal responsibility, act[s] as the means by which power is maintained over populations under late liberal capitalism’. At the same time, however, his article complicates this narrative by showing us that the success of MBCBT’s transmission within British culture hinged upon enchantment and charisma, through a reading of Weber that draws out how the ‘emotional force of religious belief continued to animate bureaucratised and rationalised settings’.

The intertwined relationship between advanced liberal polities and the knowledge and practice of the psy-disciplines appears to be self-evident. But articles in these special issues also bolster the emerging historiographical consensus among scholars of the Soviet sphere of influence, who show that they also had their role to play in the governance of socialist societies (Brokman, 2018; Eghigian, 2015; Gjuričová, 2018; Lišková, 2018; Marks, 2018; Raikhel and Bemme, 2016; Reich, 2018). This suggests neoliberalism has more in common with its antitheses than we might first suspect. Yet while the psy-disciplines, and psychotherapy specifically, are an apparently ubiquitous symptom of modernity, we need to pay closer attention to their heterogeneity. Psychoanalysis and MBCBT, for example, share more common ground with literary interpretation and yoga respectively than they perhaps do with each other. Stepping back and looking at the special issues’ contributions across this wide geographical terrain, one might be tempted towards a triumphalist narrative of globalising therapeutic hegemony – psychotherapy shows no sign of losing its sway in 21st-century culture. But a more forensic inspection shows that we are often talking about wildly different practices, epistemologies and ontologies. The terms ‘psy-disciplines’ and indeed ‘psychotherapy’ obfuscate as much as they reveal. As with all histories of science, then, the task remains to delineate how and why particular approaches emerge, prevail or fall out of use, what this tells us about their locales and the wider constellations of which they form a part.

## Note

The author would like to thank Rachael Rosner and Nadine Weidman for conceiving of the idea for these parallel issues of History of the Human Sciences and History of Psychology, and for a fruitful collaboration; as well as Felicity Callard, Rhodri Hayward, Annette Mühlberger, Daniel Pick, Robert Priest, Sonu Shamdasani and Sarah Thompson.

1. See, for example, a recent conference on ‘Psychoanalysis and in the Shadow of War and Holocaust: Poles – Germans – Jews’ and the work of the Rafael Institute in Prague, which emerged from links between psychotherapists and the Charter 77 dissident human rights movement [http://www.rafaelinstitut.cz/o_nas.html]
